# Disposal of unclaimed remains in Chinese medical institutions: power-responsibility conflicts, standardization deficits, and legal regulation

**DOI:** 10.3389/fpubh.2026.1897828

**Published:** 2026-09-22

**Authors:** Nanxi Chen, Hailiang Li

**Affiliations:** The First People’s Hospital of Zunyi, Zunyi, China

**Keywords:** collaborative governance, conflict of power and responsibility, legal regulation, medical institution, public health security, standardization, unclaimed remains

## Abstract

**Background:**

The disposal of unclaimed remains is an interdisciplinary governance issue at the intersection of public health security and funeral governance in China, involving health, public security, civil affairs and finance authorities. Medical institutions, as the frontline implementers of temporary storage, death-information verification and custody handover, bear obligations that are not matched by corresponding administrative authority. This study addresses three questions: what legal and procedural norms govern the disposal of unclaimed remains in Chinese medical institutions; why power-responsibility conflicts arise and persist; and how they can be resolved through legal regulation.

**Methods:**

A triangulated qualitative design was adopted. First, national laws and administrative regulations (including the Regulations on Funeral and Interment Administration, as revised in 2025, and the Civil Code) and eight local administrative measures were analyzed through structured policy-text coding. Second, documented disposal cases and public statistics from media investigations and field-research reports (2001–2025) were examined. Third, institutional rules across regions were compared along five dimensions: publicity cycle, storage time limit, ash retention period, financial guarantee and departmental responsibility allocation.

**Results:**

Four structural problems were identified: (1) fragmented legal norms with substantial provincial variation and no unified national implementation standards; (2) nominal cross-departmental collaboration without enforceable linkage mechanisms; (3) a severe power-responsibility imbalance that shifts storage costs, public health exposure and legal liability onto medical institutions; and (4) inconsistent judicial adjudication of disposal disputes. These problems are explained as the outcome of uneven standardization in China’s funeral governance, in which local standards proliferate while universal, enforceable national standards remain absent.

**Conclusion:**

The 2025 revision of the Regulations on Funeral and Interment Administration provides a window for institutional reform. We propose five integrated strategies – unified national standards, a multi-departmental collaborative mechanism, dedicated public funding, judicial guidelines and hospital-level internal control – and conclude that legal regulation of unclaimed-remains disposal should be understood as a standardization project that balances public health security, deceased dignity and institutional equity.

## 1 Introduction

### 1.1 Background

Research Background The disposal of unclaimed remains is a common public governance issue worldwide, which is closely related to public health security, personal dignity of the deceased, legitimate rights and interests of relatives, and social public order stability. Unclaimed remains in medical scenarios refer to the remains of patients who die in medical institutions and cannot complete cremation and burial procedures within the statutory time limit due to unidentified identity, long-term missing close relatives, explicit refusal of family members to go through funeral procedures, or detention in hospitals caused by medical disputes and criminal judicial investigations (1). Medical institutions are legally obligated to undertake temporary remains storage, issuance of medical death certificates, daily mortuary custody, preliminary information verification and remains handover and filing, but have no independent administrative authority for disposal approval, cremation decision and final ash disposal of remains (2).

In recent years, China’s funeral management system has been continuously optimized, and provinces and cities have successively issued special administrative measures for unclaimed remains, further standardizing hospital mortuary management and cross-departmental remains handover procedures (3). However, statistical data from civil affairs departments show that a large number of unclaimed remains have been detained in medical institutions and funeral parlors for a long time, which not only occupies medical hardware resources and public funeral service resources, increases the operational burden of medical institutions, but also easily causes microbial breeding due to remains decay and significantly raises potential risks of nosocomial infection and regional public health hazards (4). From the perspective of global public health prevention and control, non-standard disposal of remains in medical institutions is an important inducement for pathogen aerosol transmission and cross-infection. Standardized whole-process remains management is the core link for routine public health prevention and control and emergency response to public health emergencies in medical institutions (5).

Practice of major public health emergencies including SARS, Ebola and COVID-19 has confirmed that the number of in-hospital deaths surges during pandemics. If detained remains fail to be disinfected, stored and disposed of in a timely and standardized manner, the risks of nosocomial infection and social public health crisis will be further amplified (6). At present, the governance of unclaimed remains in China presents prominent characteristics of institutional fragmentation, localized standards and decentralized performance of duties, forming a governance paradox that medical institutions undertake the obligation of territorial custody but have no disposal decision-making power, while multiple departments conduct separate supervision without a responsible subject for final accountability. Systematic reconstruction from the perspectives of legal regulation, power-responsibility division, coordination mechanism and financial guarantee is urgently required.

To situate this problem for an international readership, three features of the Chinese context require emphasis. First, China’s funeral governance is layered: the State Council’s Regulations on Funeral and Interment Administration provide the national framework, departmental circulars issued by the Ministry of Civil Affairs supply administrative guidance, and provincial and municipal governments enact local administrative measures that specify operational details (1, 3, 7). The Regulations were comprehensively revised in 2025 (State Council Order No. 824, effective 30 March 2026), explicitly assigning health authorities supervisory responsibility for mortuary premises in medical institutions and requiring local finance departments to include basic funeral service expenditure in their budgets; these changes directly affect the institutional position of hospitals examined in this paper (3, 8). Second, because the national regulation leaves implementation standards to local governments, provinces and cities differ markedly in claim publicity cycles (60–90 days), maximum storage periods, ash retention periods and funding arrangements (4, 9–12). Third, these local differences are not merely administrative details; they generate unequal treatment of the deceased and unequal legal exposure for medical institutions across regions, which is precisely the standardization deficit analyzed below.

This study adopts the sociology of standards and standardization developed by Timmermans and Epstein (13) and Timmermans and Berg (14) as its integrative analytical lens. Standardization is understood not as the mechanical imposition of uniform rules but as a contested process through which actors negotiate the locus, specificity and enforcement of standards. Applied to unclaimed-remains disposal, this lens makes visible three questions that the existing literature leaves implicit: where standards are set (national versus local), how specific they are (procedural versus outcome-oriented), and how they are enforced (administrative accountability versus judicial review). The framework is elaborated in Section 2.3 and used to interpret the findings in Section 6.

### 1.2 Literature review and research gaps

#### 1.2.1 Overseas research status

Foreign academic research on unclaimed remains disposal mainly forms four research veins: protection of the deceased’s ethical dignity, definition of public attributes of funeral services, risk prevention and control of remains in medical institutions, and legal protection of personal information of the deceased (15, 16). In the field of public health management, systematic studies have confirmed that non-standard temporary storage, disinfection and handover procedures of remains in medical institutions will significantly increase the pathogen exposure risks of medical staff, mortuary administrators and in-hospital patients, clarifying the rigid necessity of standardized operational specifications for remains disposal (5). Finegan et al. (17) formulated special management guidelines for hospital remains during global public health emergencies, pointing out that the lack of unified implementation standards for the emergency disposal of infectious unclaimed remains is a universal governance shortcoming worldwide.

In the field of funeral governance, foreign scholars focus on the logic of cross-organizational collaborative governance, confirming that clear division of power and responsibility, information resource sharing and joint collaboration mechanism can effectively solve practical difficulties such as insufficient supply of funeral services and long-term backlog of detained remains (15). From the perspective of legal regulation, Zadyraka proposed that the collection, publicity and subsequent disposal of the deceased’s personal information should balance the needs of public governance with the personal dignity of the deceased and privacy rights and interests of family members, and improved administrative procedural norms are the prerequisite for legalized remains disposal (16). Goyet et al. (18) corrected the public misunderstanding that remains may spread epidemics, verifying that standardized closed storage, regular disinfection and closed-loop management can effectively eliminate public panic and hidden dangers of public health governance. Meanwhile, medical and funeral practitioners belong to high-risk groups exposed to remains pathogens, and standardized protection and procedural control are the key to occupational health protection (19).

#### 1.2.2 Domestic research status

Existing domestic studies mostly focus on macro revision of funeral legislation, reform of civil affairs management system and summary of local disposal experience, mainly taking government administrative departments as the research subject and analyzing institutional defects of the funeral governance system from the perspective of administrative management (9, 11). The existing achievements have initially constructed the basic theoretical framework for the governance of unclaimed remains, but most studies remain at the top-level policy design level, paying insufficient attention to the front-line practical scenarios, operational pressure and legal risks of medical institutions (10).

#### 1.2.3 Research gaps

The existing research has three core shortcomings. First, few studies take medical institutions as an independent research subject, so the custody obligations, operational costs and legal accountability risks borne by hospitals in frontline disposal scenarios remain underexamined. Second, most studies are confined to the textual interpretation of legal norms; they neither map cross-departmental power-responsibility conflicts in a structured way nor verify them against documented cases, which leaves proposed countermeasures overly abstract and difficult to implement. Third, mature international research on public health remains management, cross-organizational collaborative governance and deceased-person privacy protection has not been fully integrated into China’s localized institutional design. By adopting the micro perspective of medical institutions, this paper addresses these gaps in both perspective and empirical grounding.

### 1.3 Research significance

#### 1.3.1 Theoretical significance

This study makes three theoretical contributions. First, it extends the sociology of standards and standardization (13, 14) into the domain of Chinese funeral governance, showing that the chronic detention of unclaimed remains is best understood as a standardization deficit – a proliferation of local standards without a universal, enforceable national standard – rather than as a mere administrative backlog. Second, it refines the concept of power-responsibility imbalance in public service delivery: medical institutions occupy a ‘duty-bearing without decision-making’ position in which storage and infection-prevention obligations are legally fixed while disposal authority is located in other agencies, a configuration that predictably shifts risk to the frontline actor (3, 8). Third, it specifies the distinct analytical role of each theoretical pillar – standardization theory as the integrative lens, collaborative governance theory as the mechanism design, public risk governance theory as the consequence analysis, and public interest theory as the normative foundation – thereby avoiding the conflation of multiple theoretical claims that has weakened prior work. In doing so, the study provides a transferable analytical framework for similar standardization problems in health-related public services.

#### 1.3.2 Practical significance

Practically, this paper maps the institutional conflicts, process bottlenecks and risk sources in the disposal of unclaimed remains in medical scenarios, and traces them to their institutional roots. Drawing on international models of remains storage, disinfection protection and cross-organizational collaboration (17, 19–21), it constructs a whole-process standardized disposal specification and a multi-departmental collaborative supervision mechanism. These instruments can reduce the operational costs, administrative penalties, civil disputes and public health infection risks of medical institutions, and support the institutionalization and standardization of medical-funeral governance in China.

### 1.4 Research methods

This study employs a triangulated qualitative research design combining three methods – policy text analysis, case empirical analysis and comparative institutional analysis. The analytical framework integrates standardization theory (13, 14) with collaborative governance theory (15), risk governance theory and public interest theory; the operationalization of each method is described below.

#### 1.4.1 Data sources

Four categories of documents constitute the primary data. (1) National legislation and administrative regulations: the Regulations on Funeral and Interment Administration (as amended in 2012 and comprehensively revised in 2025, State Council Order No. 824, 3), the Civil Code of the People’s Republic of China (2020), the Law on the Prevention and Control of Infectious Diseases, and the Ministry of Civil Affairs’ Circular on Further Standardizing the Disposal of Remains and Ashes (Min Ban [2024] No. 27, 2). (2) Local administrative measures: eight provincial or municipal instruments enacted by Baotou, Gansu, Zhengzhou, Jiangmen, Dongguan, Chongqing and Kunming, covering publicity cycles, storage limits, ash retention and funding arrangements (1, 4, 7, 9–12). (3) Public statistics and investigative reports: published statistics of civil affairs departments (e.g., Gansu, Hohhot, and Dongguan) and investigative journalism by Xinhua, China News Service and Southern Weekend documenting detention durations, mortuary occupancy rates and cost burdens. (4) Academic and field-research literature: peer-reviewed studies on epidemic remains management (6, 17, 19, 21–24), international comparisons of unclaimed-body management (20) and field research on long-term detention of remains in Chinese cities (25).

#### 1.4.2 Search strategy and inclusion criteria

Documents and cases were located through three channels: official government websites and gazettes, legal databases (PKU Law; China National Knowledge Infrastructure) and public media archives. The search covered the period 2001–2025. Inclusion criteria were: (i) documents that normatively govern the identification, storage, publicity, cremation or ash disposal of unclaimed remains; and (ii) cases or statistics that report the actual detention, cost or dispute outcomes of unclaimed remains in medical institutions or funeral parlors. Exclusion criteria were: (i) documents that merely mention funeral services without specific provisions on unclaimed remains; (ii) duplicate or superseded versions of the same instrument; and (iii) unverifiable second-hand case reports lacking a citable original source. In total, 26 national and local legal documents and 12 documented case clusters were retained for analysis.

#### 1.4.3 Coding and analysis framework

All legal documents were coded along five dimensions derived from the standardization framework: claim publicity cycle, maximum storage period, ash retention period, financial guarantee mechanism and departmental responsibility allocation. Cases were coded thematically for detention duration, stated cause (unidentified identity, unreachable relatives, family abandonment or dispute), institutional consequence (cost, infection risk, administrative penalty or lawsuit) and resolution outcome. Coding was conducted independently by two researchers; disagreements were resolved through discussion with a third reviewer until full consensus was reached.

#### 1.4.4 Comparative dimensions

The comparison operates at two levels. Domestically, the eight local instruments were compared on the five coded dimensions to quantify inter-regional variation (Table 1). Internationally, China’s institutional design was benchmarked against documented models in the Asia-Pacific region (20) and against WHO and ICRC guidance for the management of dead bodies in health emergencies (6, 17), which served as reference points for identifying standardization deficits.

**Table 1:** Comparison of local standards for unclaimed remains disposal in typical provinces and cities.

Region	Claim publicity cycle	Maximum reference storage period	Ash retention period	Special financial funding
Zhengzhou	90 days	3 days, extension applicable	2 years	No unified special fund
Gansu	Minimum 60 days	3 days, strict overstay control	2 years	Partial overall financial support
Jiangmen	60 days with overdue reminder	Strictly control overstay	1 year	Appropriate local financial subsidy
Chongqing	60–90 days	Classified differentiated control	2 years	Pilot special fund establishment

#### 1.4.5 Reliability and limitations

Triangulation across the four data categories was used to corroborate each empirical claim: a statistic was accepted only when it was traceable to an identified issuing institution or publication. The design has two inherent limitations, acknowledged from the outset: the empirical base consists of public statistics and documented cases rather than primary survey data, and the local instruments analyzed are not exhaustive of all Chinese regions. These limitations are revisited in the Discussion.

### 1.5 Research innovation

#### 1.5.1 Perspective innovation

Different from traditional macro governance research led by the government, this paper takes medical institutions as the core research subject, focusing on front-line operational dilemmas and legal risks such as mortuary custody, remains handover and risk underwriting, highlighting the micro characteristics of power-responsibility imbalance.

#### 1.5.2 Content innovation

It disassembles the conflict breakpoints of power and responsibility among health, public security, civil affairs and finance departments through a structured mapping of institutional rules, and integrates international research on public health prevention and control, organizational collaboration and personal privacy protection into a unified demonstration framework.

#### 1.5.3 Application innovation

Based on current national regulations and local practical policies, it constructs an implementable whole-process standardized disposal mechanism and cross-departmental collaborative supervision system for unclaimed remains, with both institutional normative value and practical operational guidance.

## 2 Concept definition and theoretical basis

### 2.1 Concept and classification of unclaimed remains

Combined with national norms and local administrative measures, unclaimed remains in medical scenarios are defined as: remains of patients dying in territorial medical institutions who fail to complete funeral and cremation procedures within the specified time limit due to unidentified identity, missing relatives, family refusal to claim, medical disputes or judicial investigation (1, 10). They are specifically divided into four categories: ① Unidentified remains with completely unverifiable identity and no relative clues; ② Uncontactable remains with identifiable identity but unreachable close relatives; ③ Abandoned remains whose family members know the death fact but explicitly refuse to claim and undertake funeral obligations; ④ Dispute-detained remains detained in hospitals due to medical disputes, judicial appraisal and criminal cases (4). This classification conforms to China’s funeral management system and adapts to the research paradigm of international public health governance (5) (Table 2).

**Table 2:** Classification and governance characteristics of unclaimed remains in medical institutions.

Type of remains	Core definition characteristics	Major governance difficulties	Responsible relevant authorities
Unidentified Remains	Completely unverifiable identity information; no relative clues	Difficult identity verification; long claim cycle; complicated disposal approval	Public Security, Civil Affairs, Medical Institutions
Uncontactable Remains	Verifiable identity but unreachable close relatives	High cost of information investigation; long publicity period	Public Security, Civil Affairs, Medical Institutions
Abandoned Remains	Family members aware of death but refuse to claim and bear funeral expenses	Ethical disputes; unborne expenses; public opinion risks	Civil Affairs, Medical Institutions
Dispute-detained Remains	Disposal rejected due to medical disputes, judicial appraisal or criminal cases	Slow judicial intervention; long-term occupation of mortuary resources; high dispute risks	Health, Justice, Public Security, Medical Institutions

### 2.2 Institutional power and responsibility boundary of medical institutions

In accordance with the Regulations on Funeral and Interment Administration, medical institutions are responsible for issuing medical death certificates for in-hospital natural deaths, and undertaking public welfare temporary storage of mortuary remains, basic information verification, daily custody and cross-departmental remains handover (3). The regulations clearly stipulate that hospital mortuaries are prohibited from illegal outsourcing and commercial funeral services, retaining only the public welfare function of temporary storage (3). For unnatural deaths, medical institutions shall immediately report to the public security department and assist in remains on-site investigation and identity information collection (1).

In terms of authority, medical institutions have no independent authority over cremation approval, disposal decision-making and ash disposal; all final disposal acts require administrative approval from public security and civil affairs departments (9). In terms of obligations, medical institutions bear long-term rigid duties including low-temperature storage, regular disinfection, infection prevention and control, and account management, producing a power-responsibility imbalance in which obligations are overloaded while administrative authority is absent. At the public health level, medical institutions undertake the core obligations of remains disinfection, environmental sterilization and nosocomial infection prevention and control; non-standard management can directly induce public health safety incidents (5). At the civil-law level, Article 994 of the Civil Code protects the personality interests of deceased persons – including their name, image, reputation, honor, privacy and remains – and entitles spouses, children and parents to seek civil remedies for infringement (8); this provision converts dignified custody of remains into a legally enforceable duty of medical institutions. Mortuary staff and medical personnel belong to high-risk groups exposed to remains pathogens, and standardized procedural management is the basic guarantee for occupational safety and public health prevention and control (19, 23).

### 2.3 Core theoretical basis

#### 2.3.1 Standardization theory

Standardization theory, as developed in the sociology of science and medicine, examines how standards are created, diffused and contested, and why some standards become universal while others remain local (13, 14). Timmermans and Epstein distinguish four dimensions of any standard: its locus (who sets it), its specificity (how precisely it prescribes action), its enforcement (how compliance is secured) and its scope of application (13). Applied to unclaimed-remains disposal, the theory predicts that where national standards remain abstract, local actors will fill the gap with divergent operational rules, generating unequal treatment and diffuse accountability. This prediction matches the observed fragmentation of China’s funeral governance and provides the integrative lens through which the three supporting theories below are applied: collaborative governance theory addresses the mechanism of standard-setting and enforcement; risk governance theory addresses the consequences of standard failure; and public interest theory addresses the normative justification for public standards.

#### 2.3.2 Collaborative governance theory

Collaborative governance theory emphasizes cross-departmental resource integration, division of power and responsibility and joint collaboration, which is the core logic to solve public governance problems involving multiple subjects (15). The disposal of unclaimed remains involves multiple administrative authorities including health, public security, civil affairs and finance. Independent performance of duties by a single department is prone to regulatory loopholes and buck-passing. Through multi-subject information sharing, process connection and shared power and responsibility, a closed-loop whole-process management from registration, verification and publicity to cremation and ash placement can be realized, making up for the service capacity shortcomings of a single subject.

#### 2.3.3 Risk governance theory

Public risk governance requires advance prevention, source control and whole-process management. Long-term over-standard storage of unclaimed remains can lead to remains decay and prolonged pathogen survival, breeding risks of nosocomial infection, operational loss and legal disputes (5, 24). Non-standard disposal may also inflict psychological harm on medical staff and family members. Occupational infection studies demonstrate that funeral and mortuary workers face elevated exposure to infectious remains, confirming that deficient storage and protection standards are a common public health governance gap (23, 24). Scientific risk governance can therefore contain multi-dimensional hazards spanning public health, operational management and social ethics (22).

#### 2.3.4 Public interest theory

Funeral services belong to basic public welfare services. The disposal of unclaimed remains is an important part of social public governance, rather than a single internal management affair of medical institutions (11). According to the public interest theory, the government shall undertake the responsibilities of institutional supply, financial underwriting and cost guarantee to safeguard social public interests. Meanwhile, remains governance needs to balance public safety, social ethics and privacy rights of the deceased, and protect the personal dignity and legitimate rights and interests of the deceased and their close relatives while achieving public governance goals (16).

## 3 Current situation and empirical analysis

### 3.1 Standard disposal process in domestic medical institutions

Combined with national regulations and local administrative norms, the disposal of unclaimed remains is divided into four core links:

#### 3.1.1 Preliminary verification and registration

Medical institutions issue death certificates for natural deaths in hospital; report unnatural deaths to public security departments in a timely manner for investigation and verification, and complete basic information account registration (1).

#### 3.1.2 Temporary remains storage

The conventional storage period of hospital mortuaries shall not exceed 3 days in principle; extension procedures shall be handled specially for exceptional cases. Low-temperature refrigeration at 2 °C ~ 4 °C and sealed packaging are adopted to inhibit remains decay and block pathogen transmission (3, 5).

#### 3.1.3 Information publicity and verification

The public security department is responsible for identity information comparison and relative investigation; the civil affairs department releases claim publicity with a cycle of 60 ~ 90 days in most regions (4, 10). Kunming’s Administrative Measures illustrate a complete verification sequence: the public security organ conducts an on-site examination, samples and tests the remains, issues a death certificate for unidentified remains, and publishes a claim notice for 15 days; upon expiry without a claim, the county-level civil affairs department arranges disposal (7). The personal information of the deceased shall be strictly controlled during the publicity period to prevent improper privacy disclosure (8, 16).

#### 3.1.4 Cremation and ash disposal

If no valid claim is made after the publicity period expires, the competent department shall issue a disposal official reply and the funeral parlor conducts unified cremation. Ashes shall be retained for 1 ~ 2 years in accordance with regulations, and public welfare ecological burial shall be implemented if unclaimed upon expiration (12) (Figure 1).

**Figure 1:**
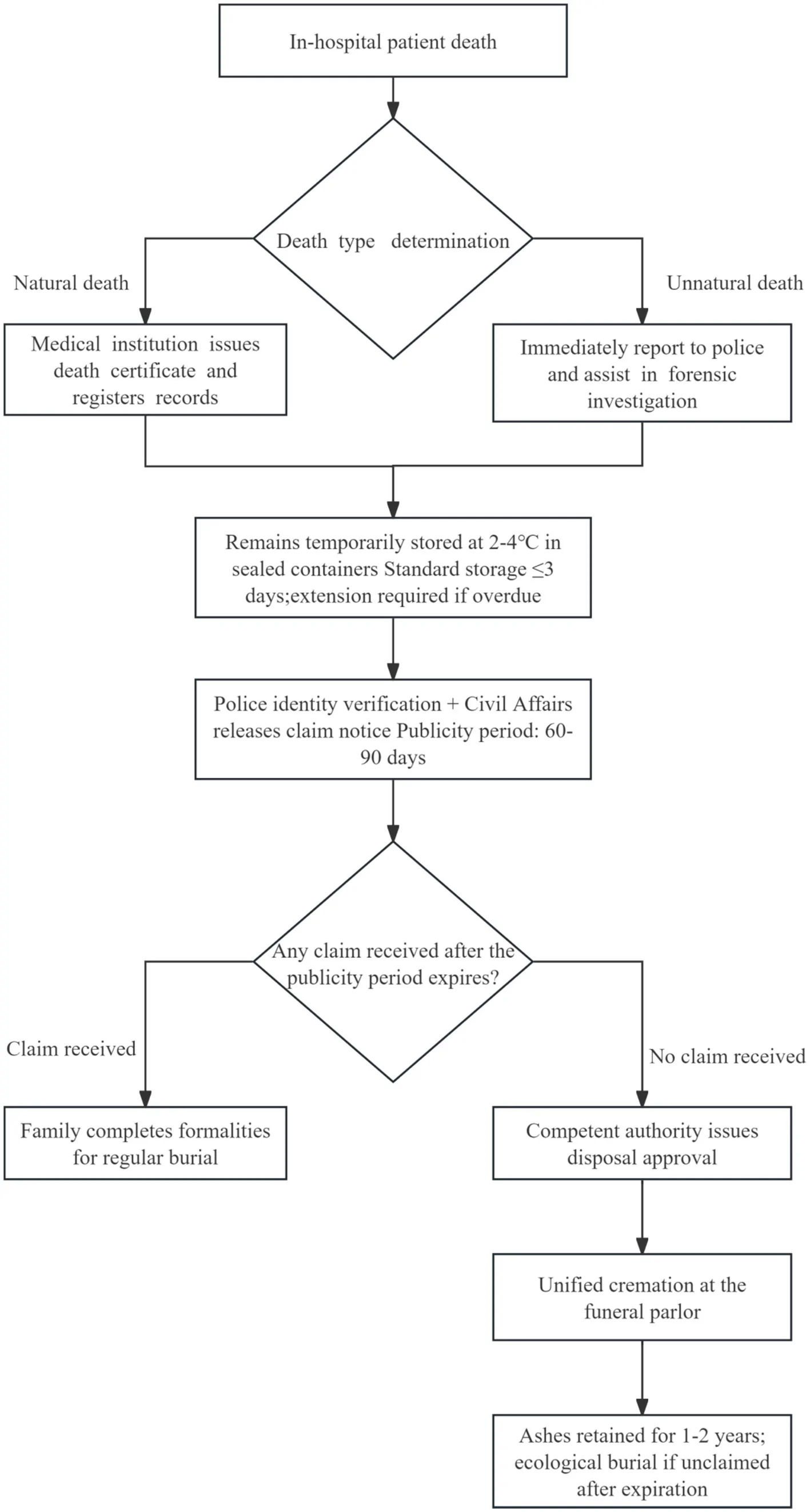
Whole-process disposal flowchart of unclaimed remains in medical institutions.

### 3.2 Empirical dilemma of practical disposal

Long-term detention of unclaimed remains in medical institutions is well documented across China. In Hohhot (Inner Mongolia), an investigation by Xinhua found that 135 of the 197 mortuary cabinets in the city’s medical institutions were occupied by unclaimed remains, some stored for over 20 years; the accumulated storage fees for a single body reached 300,000 yuan at prevailing rates (25). Similar patterns were reported in Shenyang, where about 110 of 170 mortuary cabinets in one funeral home were occupied by long-term detained remains, and in Xiangtan (Hunan), where nearly half of the 15 cabinets of a central hospital were occupied by unclaimed remains, some dating from 2007 (25).

The scale of the problem is geographically uneven but widespread. Guangzhou’s funeral parlor reportedly received 1,300–1,400 bodies per year that exceeded the claim period, reflecting the concentration of a ‘floating population’ in large cities (25). Data from the civil affairs department of Gansu Province show that local funeral parlors accumulated 54 long-term detained unclaimed remains, with accumulated over-standard storage costs of nearly 10 million yuan (4). Industry statistics from Dongguan show that the stock of unclaimed remains increases year by year and the longest detention exceeds 16 years, continuously occupying mortuary capacity, refrigeration equipment and public funeral resources (9).

A field-research-based account from the First Affiliated Hospital of Kunming Medical University illustrates the operational reality behind these statistics. As of 2021, 24 unclaimed remains occupied a mortuary designed for 12 bodies; the oldest had been stored for 16 years, and hospital staff had to publish claim notices, coordinate forensic examination by the public security organ, and search for relatives at their own initiative and expense (7, 25). Such cases show that the procedural gap is not a lack of goodwill among frontline staff but a structural absence of clear authority, funding and handover standards.

In operational practice, most regions provide no special financial subsidy for the disposal of unclaimed remains; equipment operation and maintenance, personnel duty and environmental disinfection costs are borne by the medical institutions themselves (10). Long-term non-standard storage of remains readily breeds microorganisms and increases the risk of nosocomial cross-infection; pathogen aerosol transmission caused by non-standard handling is an important inducement of nosocomial infection (24). Once remains decay, procedural irregularities or information disclosure problems occur, medical institutions independently face administrative penalties and civil compensation liability (11).

### 3.3 Multiple governance risks of medical institutions

#### 3.3.1 Operational cost risk

Long-term remains refrigeration, equipment depreciation, personnel custody and regular disinfection form continuous expenditure. The lack of financial settlement and compensation mechanism squeezes the normal operating funds of medical institutions for a long time (10).

#### 3.3.2 Public health security risk

Overdue remains decay, non-standard disinfection and insufficient personnel protection easily cause pathogen transmission and occupational exposure, inducing nosocomial cross-infection (5, 24).

#### 3.3.3 Administrative penalty risk

In accordance with the Regulations on Funeral and Interment Administration, any irregularity in remains storage, reporting, handover and mortuary management will result in warnings, fines and other administrative penalties. Blurred power-responsibility boundaries easily lead to passive irregularities of medical institutions (3).

#### 3.3.4 Civil dispute and public opinion risk

Family members claim compensation on the ground of unauthorized disposal after remains disposal; improper information publicity triggers privacy infringement disputes (16). Disposal methods violating social ethics easily cause online public opinion and damage the reputation of medical institutions (18) (Figure 2).

**Figure 2:**
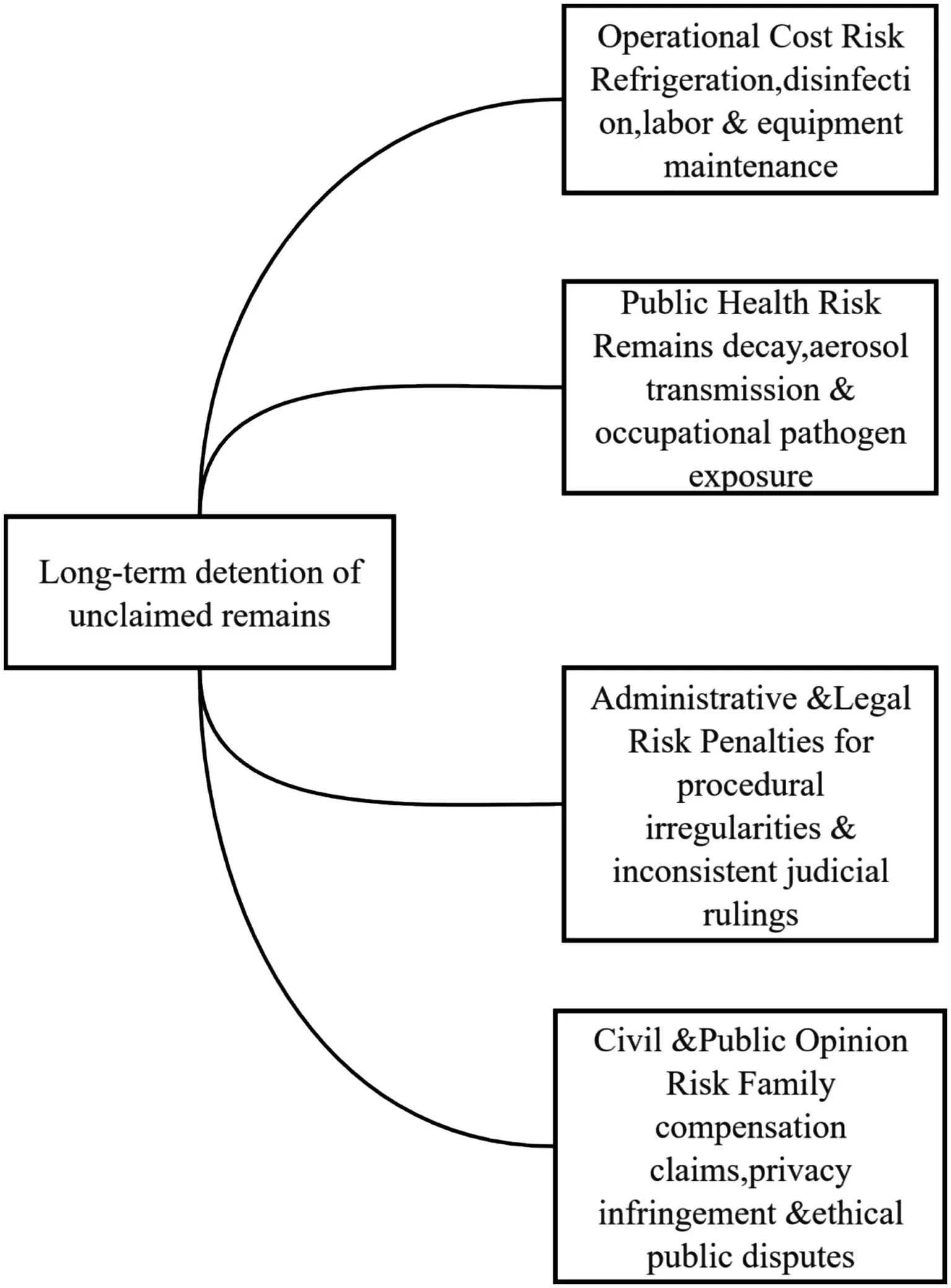
Risk transmission structure of unclaimed remains governance in medical institutions.

## 4 Conflict of power and responsibility and institutional causes

### 4.1 Vague and conflicting legal norms

China’s legal framework for unclaimed-remains disposal is layered but incomplete. At the level of national law, the Civil Code (2020) protects the personality interests of deceased persons, including their remains, and the Law on the Prevention and Control of Infectious Diseases requires sanitary handling of bodies during epidemics (8). At the administrative-regulation level, the Regulations on Funeral and Interment Administration (2012; comprehensively revised in 2025 by State Council Order No. 824, effective 30 March 2026) constitute the core national instrument; the 2025 revision adds provisions on the supervision of hospital mortuary premises by health authorities and on the inclusion of basic funeral service expenditure in local finance budgets (3). At the departmental-rule level, the Ministry of Civil Affairs has issued circulars on the standardized disposal of remains and ashes (2). Below these layers, provincial and municipal governments enact administrative measures that actually determine daily practice (1, 4, 7, 9–12).

Despite this layered structure, the core implementation standards vary significantly across regions. In terms of publicity cycle, Zhengzhou sets 90 days, Gansu a minimum of 60 days, and Jiangmen adds a 60-day overdue reminder mechanism (4, 10, 11); the ash retention period adopts multiple local standards of 1, 2 and 3 years (12). The 2025 revision leaves these operational details to local rules, so the standardization deficit persists. Moreover, the current legal system lacks special procedural norms for the verification, publicity and destruction of the deceased’s personal information, leaving institutional gaps in dignity and privacy protection (8, 16). Nor are there dedicated clauses for the emergency disposal of infectious unclaimed remains, which limits adaptability to public health emergencies (6, 17).

### 4.2 Fragmented cross-departmental governance

The disposal of unclaimed remains involves four main authorities: health, public security, civil affairs and finance, with clear division of duties but lack of normalized linkage mechanism. Health authorities are responsible for front-line remains management and death certificate issuance; public security undertakes identity verification and unnatural death investigation; civil affairs leads publicity approval and cremation placement; finance is responsible for fund guarantee. In practice, all departments perform duties independently with isolated information and fragmented supervision. Compared with mature foreign cross-organizational collaborative models, China lacks fixed communication mechanisms and responsibility connection nodes. For disputed remains with blurred power-responsibility boundaries, superficial supervision and substantive absence are common, directly resulting in long-term remains detention and seriously delayed disposal.

### 4.3 Severe imbalance of power and responsibility in medical institutions

As the front-line implementer, medical institutions undertake whole-process basic obligations including remains storage, information registration, regular disinfection, risk prevention and control and cross-departmental docking. Once disposal accidents, public health hidden dangers or family claims occur, they need to independently bear all legal and management liabilities, as well as heavy public health infection prevention obligations. However, in terms of administrative authority, medical institutions have no power to initiate publicity, approve disposal or decide cremation, relying entirely on approval from external departments. The serious mismatch of power and responsibility forces medical institutions to passively bear various governance risks.

### 4.4 Inconsistent judicial adjudication standards

Due to the lack of unified national judicial guidelines, the adjudication scales of courts in different regions on remains disposal disputes are differentiated. In similar power-responsibility disputes, some courts hold that medical institutions shall be liable for compensation for failing to fulfill custody obligations; others attribute the absence of departmental collaboration to administrative governance defects and exempt medical institutions from liability. There are no unified judicial judgment rules for privacy infringement disputes over the deceased’s personal information, aggravating the uncertainty of legal risks of medical institutions.

### 4.5 Summary of institutional root causes

The existing legal regulatory framework remains defective: uniform nationwide disposal standards are absent, alongside insufficient normative provisions governing emergency public health response and the privacy protection of deceased individuals. Cross-departmental collaborative governance is largely nominal, as institutional mechanisms for information exchange, coordinated oversight and administrative accountability have not been formally established, resulting in conspicuous fragmentation across the whole governance chain. Moreover, the financial safeguard system is underdeveloped. Without earmarked special funds and standardized cost-sharing rules for unclaimed remains disposal, public governance expenditures are unduly shifted onto medical institutions. Finally, judicial remedy channels are incomplete, leaving no mature exemption provisions or dispute-settling mechanisms to shield hospitals from secondary liabilities incurred due to dereliction of duty by relevant administrative authorities.

## 5 Optimization strategy of legal governance

### 5.1 Improve legal system and unify national governance standards

Building on the 2025 revision of the Regulations on Funeral and Interment Administration, the central government should formulate unified national detailed rules for the disposal of unclaimed remains, benchmarking uniform standards for storage time limits, claim publicity cycles, ash retention periods and emergency disposal procedures (3). The power-responsibility list of medical institutions should be refined, defining legal obligations, operational boundaries and exemption circumstances for remains detention caused by administrative delay. International public health norms for infectious remains and legal rules for deceased-person information protection should be integrated, and special clauses on remains disinfection, nosocomial infection prevention and classified information publicity should be added, so as to eliminate local divergence and realize the standardization of whole-process disposal (6, 13, 17).

### 5.2 Construct multi-departmental collaborative governance mechanism

Establish a normalized joint meeting system of health, public security, civil affairs and finance, clarifying the fixed division of labor led by civil affairs, verified by public security, custody undertaken by health, and fund guaranteed by finance. Build a provincial unified funeral remains information management platform to realize real-time sharing and whole-process traceability of death information, storage status, identity verification, publicity progress and disposal approval. Learn from foreign experience in cross-organizational collaborative governance of funeral services, establish a cross-departmental supervision and accountability mechanism, solve the governance dilemma of multiple supervision without final accountability, and form a closed-loop whole-process supervision system.

### 5.3 Set up special funds and establish cost-sharing system

The 2025 revision of the Regulations on Funeral and Interment Administration requires local finance departments to include basic funeral service expenditure in their budgets; this study recommends that the disposal of unclaimed remains be added to the scope of such expenditure (3). Dedicated public welfare funeral funds should be established to cover remains storage, disinfection, transportation, cremation and ecological burial. Differentiated cost-sharing standards should be formulated according to remains type (unidentified, abandoned, medical-dispute and criminal-case remains), and a normalized mechanism of advance payment by medical institutions with regular financial settlement should be established, preserving the public welfare attribute of funeral services and relieving the operational burden of medical institutions (10).

### 5.4 Optimize risk prevention and control and judicial remedy system

Construct a whole-process internal risk control system for medical institutions, standardize remains registration, handover signature, image retention and file archiving system to achieve traceable whole-process disposal. Strictly implement standardized operations such as sealed remains packaging, classified storage, regular disinfection and occupational protection to block nosocomial infection risks from the source. Issue judicial guidelines for similar cases, unify the adjudication scale of remains disposal disputes, clarify the exemption scope of medical institutions caused by absent performance of duties of other departments; formulate special judicial judgment standards for privacy infringement disputes of the deceased, improve judicial relief channels for medical institutions, and avoid passive liability.

### 5.5 Standardize internal operational system of medical institutions

Medical institutions shall formulate special internal management regulations for unclaimed remains, improving whole-process norms including mortuary management, death verification and registration, abnormal situation reporting, extension application and remains handover. Emergency disposal plans for suspected infectious and highly decayed remains shall be developed in accordance with domestic and international public health standards (5, 17, 21). The collection, storage, publicity and destruction of the deceased’s personal information shall be strictly standardized, and the private dissemination of the deceased’s images and privacy information is prohibited, in line with Article 994 of the Civil Code and applicable personal-information rules (8, 16). A permanent archive system shall be established to enable traceability and review of the entire disposal process.

## 6 Discussion

This study set out to explain why unclaimed remains accumulate in Chinese medical institutions and how legal regulation can address the problem. The findings can be summarized as four propositions: (1) the legal framework is layered but the operational standards are fragmented; (2) inter-departmental collaboration is nominal; (3) medical institutions bear obligations without decision-making authority; and (4) judicial responses are inconsistent. Interpreted through the standardization lens (13, 14), these four propositions share a single root cause – a standardization deficit in which national rules prescribe ends (dignified, timely disposal) but delegate means (publicity cycles, storage limits, funding and accountability) to uncoordinated local actors.

The power-responsibility imbalance identified in Section 4.3 is not a peculiarity of Chinese hospitals but a structural feature of public-service arrangements in which the actor physically closest to the service user (here, the hospital mortuary) is assigned the greatest operational risk while the authority to act resides elsewhere (3, 8). International comparisons support this diagnosis: jurisdictions with explicit unclaimed-body protocols and designated agencies – such as those compared by Khoo et al. (20) across four Asia-Pacific countries – achieve shorter storage durations precisely because they institutionalize who may dispose, who pays and who is accountable. China’s 2025 revision of the Regulations takes a step in this direction by assigning health authorities supervisory responsibility for mortuary premises and requiring financial inclusion in local budgets, but it does not yet specify the operational standards that would close the loop (3).

The public health dimension of the findings deserves emphasis. Consistent with WHO and ICRC guidance and with the occupational infection literature, our case evidence indicates that prolonged, non-standardized storage elevates infection risk for hospital staff and the wider community (6, 17, 23, 24). The ‘disaster myth’ literature cautions, however, that dead bodies are rarely a source of epidemics; the realistic risk is occupational and nosocomial, which makes standardized handling and disposal procedures – rather than panic-driven measures – the appropriate policy response (18, 19).

Three implications follow for reform. First, standardization should be pursued at the level of implementation standards, not only framework law; national detailed rules should fix publicity cycles, storage limits and funding responsibilities (13). Second, the coordination mechanism should be proceduralized – joint meetings, information platforms and accountability nodes – because the current configuration of ‘multiple supervisors, no responsible subject’ reproduces fragmentation (15). Third, the financial burden should be reallocated through dedicated funds, as the 2025 revision anticipates, so that hospitals are not forced to price dignified storage against operational survival (3, 10).

The limitations of this study should be acknowledged. The empirical base rests on public statistics and documented cases rather than primary survey data, and the local instruments analyzed are not exhaustive of all Chinese regions; triangulation across sources mitigates but does not eliminate these constraints. Future research could expand the sample of medical institutions nationwide, quantify the economic cost of remains detention and the level of public health risk, and develop differentiated governance schemes for minority, foreign-related and major-epidemic scenarios, thereby further improving China’s funeral public health governance.

## 7 Conclusion

The disposal of unclaimed remains is an important part of public health security and funeral public welfare governance in China. The current governance system has prominent problems such as inconsistent legal standard implementation, fragmented cross-departmental governance, severe power-responsibility imbalance of medical institutions, absence of long-term financial guarantee and differentiated judicial adjudication scales. The root causes lie in imperfect institutional supply, absence of collaborative mechanism, unreasonable cost-sharing and blocked relief channels. To resolve the governance dilemma, efforts should be made from five dimensions: optimizing top-level legal regulation, constructing cross-departmental collaboration, establishing special financial funds, unifying judicial adjudication and improving institutional internal control, so as to build a unified national disposal system integrating international public health prevention and control and privacy protection standards, and set up a closed-loop multi-departmental linkage supervision mechanism. This study can effectively standardize the front-line disposal behaviors of medical institutions, reduce operational and legal risks, build a solid line of public health defense, and provide theoretical and practical references for improving China’s funeral public service governance system and institutional construction of similar public governance fields.

## Data Availability

The original contributions presented in the study are included in the article/supplementary material, further inquiries can be directed to the corresponding author.
